# An Obituary for Professor Dr Zainul Fadziruddin Zainuddin

**DOI:** 10.21315/mjms2019.26.5.14

**Published:** 2019-11-04

**Authors:** Zainul Fadziruddin Zubair Faramir

**Affiliations:** Department of Aerospace Engineering, Faculty of Engineering, Universiti Putra Malaysia, Serdang, Selangor, Malaysia

## Introduction

Dr Zainul Fadziruddin was born on 1 April 1961 at Kuarters Guru Pekan Rantau in Negeri Sembilan, Malaysia. He was the second child of four and the only son to Mohd Zainuddin bin Shamsuddin and Jauhariah binti Haji Nordin. His father was an army veteran who later worked at the Nestle company and his mother was a primary school teacher. When he was little, his family moved to Kampung Baru in Kuala Lumpur for several years before moving to Seksyen 14 in Petaling Jaya, Selangor, Malaysia.

**Figure:**
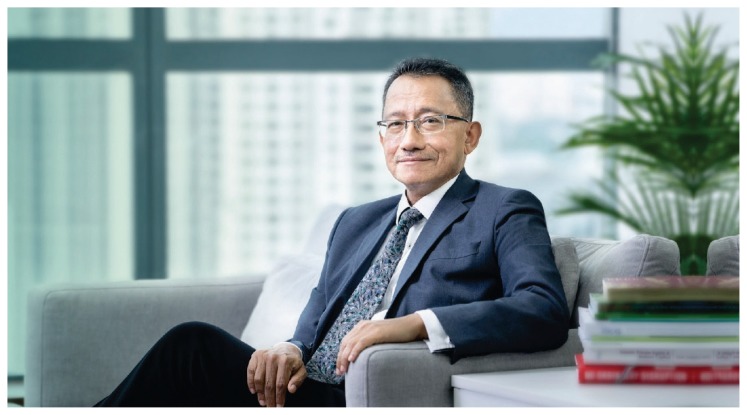
Professor Dr Zainul Fadziruddin Courtesy of Xeraya Capital, picture taken in 2018

## Achievements

Dr Zainul’s early education started at Bukit Bintang Boys Primary School in Petaling Jaya in 1968. He was among the brightest students in his class, scoring among the top three students for every examination. His excellent performance in both academics and leadership earned him the position of the Head Student for the School in 1973. In 1972, he was the only candidate from Bukit Bintang Boys Primary School to achieve 5As in the Peperiksaan Penilaian Darjah 5 and he qualified to join the prestigious Malay College Kuala Kangsar (MCKK) in 1974. In 1975, Dr Zainul discovered his passion when he was in MCKK’s library, reading a microbiology book written by a well-known French microbiologist named Louis Pasteur, the father of modern microbiology. Besides his good academic performance, he was talented in racket sports, especially table tennis. He represented MCKK in many tournaments for years and was the team leader when he was in Form 5. During his leadership period, his team once dominated the top local schools in table tennis.

He was awarded a federal government scholarship in 1979 to pursue his A-level at the Darlington College of Technology in the United Kingdom. He later completed his degree in microbiology at the University of East Anglia in the United Kingdom in 1984. During this time, he gained more exposure to and interest in microbiology at the John Innes Institute and the Food Research Institute. In addition to his excellent academic performance, he was still able to balance his life by becoming his university’s representative in table tennis. His passionate efforts in his microbiology studies were rewarded when he was offered a postgraduate study in molecular biology at the University of Surrey, Guildford in the United Kingdom. His research subject was tuberculosis and he worked under the supervision of Professor Jeremy Watson Dale.

Dr Zainul was awarded a doctor of philosophy in 1988 from the University of Surrey and returned to Malaysia to work as a lecturer at the Universiti Sains Malaysia (USM)’s School of Medical Sciences under the Medical Microbiology & Parasitology Department. His hard work in academia paid off when he received the Anugerah Saintis Muda Negara in 1993. In 1994, he was promoted to an associate professor position and to the head of the Medical Microbiology & Parasitology Department. During his tenure at USM, his research interests were in the fields of diagnostics and vaccinology in tuberculosis, cholera, malaria and enterovirus EV71. He was a leader and co-leader of numerous research projects in these areas at the university, national and international levels. Together with his colleagues and students, he published or presented more than 100 research papers at the national and international levels. Dr Zainul and his associates also managed to obtain grants from national and international sources. Despite his principal work as a lecturer, his interest in racket sports continued, with squash as his hobby.

On 1 November 1999, he led the first five team members and became the founding dean for the School of Health Sciences, USM. The first cohort of students started with just four degree programmes (Biomedicine, Forensic Science, Nursing and Dietetics) and has since expanded to 10 programmes, including Medical Radiation, Exercise and Sports Science, Audiology, Speech Pathology, Nutrition and Environment & Occupational Science. Dr Zainul led a successful journey in developing and nurturing the School of Health Sciences, which is well-known today, with the full support of other colleagues, such as Almarhum Professor Dr Syed Mohsin Syed Sahil Jamalullail, Professor Datuk Dr Asma Ismail, Professor Dr Norazmi Mohd Nor and many more, including the then-appointed Vice Chancellor, Professor Tan Sri Dzulkifli bin Abdul Razak. Since he stepped down as dean in late 2007, the School of Health Sciences has grown to almost 90 academic staff, almost 100 non-academic staff, eight programmes, and an enrolment of more than 800 undergraduate students, with thriving research and postgraduate activities. Throughout his career, Dr Zainul filed two patents together with his research team (one in 1993 for probes, kits and a method for the detection and differentiation of mycobacteria and one in 2004 for the *Vibrio cholerae* strains VCUSM1 and VCUSM4).

After years of excellence contributing to USM, on 19 December 2003, USM promoted him to full professor and he later became the director for the university’s Innovation Office, a role he served in from 2007 until 2012. During this time, he worked towards creating an internal innovation and environment ecosystem to support discovery, creativity, collaboration, intellectual property creation and the commercialisation of research output from USM. Dr Zainul helped establish USM’s APEX status, which was officially announced by the ministry on September 2008. He made tremendous efforts to develop and execute plans to move the university, national science and technology forward. Amidst his busy administrative responsibilities, he was also an editor for the *Malaysian Journal of Medical Sciences*.

In addition to his excellent academic career, Dr Zainul was a Malaysian Technology Development Centre (MTDC) board member from 2004 until early 2012. He chaired the Investment Committee for MTDC, which has created nearly 500 deal opportunities since 2006. He was appointed the senior vice president of the Malaysia Biotechnology Corporation (Biotech Corp) in 2006 and later served as a member of the Board of Biotech Corp (2009–2013). He was also the founding director of Sanggar SAINS Sdn Bhd (USM’s commercialisation arm) from 2008 until 2012 and the founding director of Innovation Xchange Malaysia Berhad (industry-university linked) from 2009 until 2012.

With vast experience in research and commercialisation, from 2 May 2012, Dr Zainul served MTDC as the director of the Business Advisory, Incubation and Nurturing Division and then as a director at the CEO’s Office. He contributed heavily to Industrial 4.0 development for Malaysia, as well as the commercialisation consultancy. Concurrently, he continued to provide immense contributions to the Board of Xeraya Capital Sdn. Bhd. (since 2011) and the Mudharabah Innovation Fund (MIF) Investment Berhad (since 2016) until his last breath on 16 August 2019 at the age of 58.

